# Platelet-related parameters at initial diagnosis for predicting the risk of chronicity in children with newly diagnosed immune thrombocytopenia

**DOI:** 10.3389/fped.2026.1821080

**Published:** 2026-06-01

**Authors:** Shuxian Zheng, Jing Liang, Wen Jiang, Ran Han, Xiaofei Guo

**Affiliations:** 1 Department of Blood Transfusion, The Sixth Affiliated Hospital of Xinjiang Medical University, Ürümqi, Xinjiang, China; 2 Department of Pharmacy, The Sixth Affiliated Hospital of Xinjiang Medical University, Ürümqi, Xinjiang, China; 3 Department of Pediatric Orthopedics, The Sixth Affiliated Hospital of Xinjiang Medical University, Ürümqi, Xinjiang, China

**Keywords:** children, chronicity, immune thrombocytopenia, platelet count, prediction

## Abstract

**Objective:**

To investigate the clinical value of platelet-related parameters and clinical characteristics at initial diagnosis in predicting the risk of progression to chronic disease in children with newly diagnosed immune thrombocytopenia (ITP), and to develop an individualized risk prediction model.

**Methods:**

This single-center retrospective cohort study consecutively enrolled pediatric patients who were newly diagnosed with primary immune thrombocytopenia at the Department of Pediatrics of our hospital between January 2022 and January 2025. According to disease course and whether the duration exceeded 12 months, patients were categorized into the chronic immune thrombocytopenia (CITP) group (*n* = 31) and the non-CITP group (*n* = 136). Platelet count (PLT), mean platelet volume (MPV), and immature platelet fraction (IPF%) were compared between groups. Independent predictors of chronicity were predicted using univariate and multivariate logistic regression analyses. A visualized nomogram was constructed based on key predictive factors.

**Results:**

Children in the CITP group were significantly older at initial diagnosis. Among platelet-related parameters, the platelet count at diagnosis was significantly lower in the CITP group, whereas mean platelet volume and immature platelet fraction were significantly higher. Multivariate analysis demonstrated that older age, lower platelet count, and higher immature platelet fraction were independent risk factors for progression from newly diagnosed ITP to chronic disease. ROC curve analysis indicated that the combined predictive model incorporating age, platelet count, and immature platelet fraction achieved the best performance, which was significantly superior to any single indicator alone. The nomogram constructed based on these variables showed good calibration, and DCA confirmed a substantial net clinical benefit across a wide range of threshold probabilities.

**Conclusion:**

Age at diagnosis, platelet count, and immature platelet fraction are effective predictors of chronicity in children with ITP. A nomogram integrating these three parameters demonstrates favorable predictive accuracy and potential clinical applicability. Nevertheless, these findings require further validation through multicenter prospective studies.

## 1 Introduction

Immune thrombocytopenia (ITP) is an acquired hemorrhagic disorder in childhood. Its core pathophysiological mechanism involves autoantibody-mediated accelerated platelet destruction, which is frequently accompanied by relatively insufficient platelet production by bone marrow megakaryocytes (1). Pediatric ITP typically presents with an acute onset, and the majority of affected children achieve spontaneous remission within 6–12 months after diagnosis, resulting in a generally favorable prognosis (2). Nevertheless, approximately 20%–30% of pediatric patients experience a disease course exceeding 12 months and progress to chronic immune thrombocytopenia, defined as ITP with persistently reduced platelet counts lasting longer than 12 months. The long-term management of chronic immune thrombocytopenia (CITP) poses substantial challenges, including impaired quality of life, cumulative treatment-related adverse effects, and increased psychological burden (3–5).

A major clinical challenge in current practice is the effective identification, at the time of initial diagnosis, of children who are at high risk of developing chronic disease. Although previous studies have explored associations between clinical factors, such as age, sex, and preceding infection, and the risk of chronicity, the reported findings remain inconsistent, and the predictive performance of these factors is limited (6–8). In recent years, advances in fully automated hematology analyzers have enabled routine acquisition of a series of platelet-related parameters except for conventional platelet count (PLT). These parameters include immature platelet fraction (IPF), mean platelet volume (MPV), and platelet distribution width (PDW) (9–12). By reflecting platelet size, heterogeneity, and the rate of newly released platelets from the bone marrow, respectively, these indices provide novel insights into the dynamic balance between platelet destruction and production. However, existing evidence regarding the predictive value of these parameters for chronic progression in pediatric ITP is heterogeneous. Most studies are limited by small sample sizes or univariate analyses, and there remains a lack of systematic multi-parameter evaluation and clinically applicable individualized prediction tools.

Therefore, we aim to systematically assess the value of platelet-related parameters at initial diagnosis for the risk of chronicity in children with newly diagnosed immune thrombocytopenia through a retrospective cohort analysis. Independent predictors were identified using multivariate analysis, followed by the development and validation of a combined prediction model integrating key clinical characteristics and laboratory indices, presented as a visualized nomogram. This approach seeks to support individualized follow-up and intervention strategies.

## 2 Materials and methods

### 2.1 Study design

This study was designed as a single-center retrospective cohort study. The study protocol was reviewed and approved by the Medical Ethics Committee of our hospital (Approval Number: LFYLLSC20250530-002). Owing to the retrospective nature of the study, only de-identified clinical data were used, and no interventions were applied that could influence patient diagnosis or treatment. Therefore, the requirement for informed consent was waived. All study procedures were conducted in strict accordance with the principles of the Declaration of Helsinki (13).

### 2.2 Study population

Pediatric patients who were newly diagnosed with primary ITP at the Department of Pediatrics of our hospital between January 2022 and January 2025 were consecutively enrolled.

The inclusion criteria were as follows: (1) age between 1 and 14 years; (2) fulfillment of the diagnostic criteria for newly diagnosed primary ITP according to the American Society of Hematology guidelines (14); (3) completion of a comprehensive complete blood count, including platelet-related parameters, at our hospital laboratory prior to diagnosis and initiation of any treatment; (4) regular follow-up at our hospital after diagnosis, with a minimum 12-month follow-up and clearly documented disease outcomes. Individuals with the following conditions were excluded: (1) secondary thrombocytopenia; (2) receipt of any treatment prior to initial diagnosis that could potentially affect platelet count or platelet function; (3) coexistence of other hematological disorders; (4) incomplete clinical or laboratory data.

During the study period, patients received routine clinical management according to contemporary pediatric ITP practice recommendations and physician judgment. First-line therapies mainly included intravenous immunoglobulin (IVIG) and/or corticosteroids, whereas TPO receptor agonists (TPO-RAs) were administered in selected patients with persistent or refractory thrombocytopenia during follow-up.

### 2.3 Outcome definition

According to the International Working Group consensus and clinical practice guidelines, grouping was performed based on platelet count and clinical status at 12 months after diagnosis (14).

CITP group: Persistent thrombocytopenia, defined as a platelet count <100 × 10⁹/L, lasting for more than 12 months.

Patients in the non-CITP group (control group) met either of the following criteria: (1) platelet counts that spontaneously or following initial treatment remained ≥100 × 10⁹/L within 12 months without the need for ongoing therapy; (2) disease duration of less than 12 months, but with stable normalization of platelet counts (≥100 × 10⁹/L) maintained for at least 3 months.

### 2.4 Observational variables and data collection

Data were extracted and cross-validated by two investigators using the electronic medical record system and laboratory information system.

Baseline clinical characteristics: Sex, age, and body mass index (BMI) at initial diagnosis were recorded. Treatment-related variables, including the use of intravenous immunoglobulin (IVIG), corticosteroids, Thrombopoietin Receptor Agonists (TPO-RAs) during the initial disease course, were also collected from medical records.

Laboratory parameters: Results from the first pre-treatment complete blood count obtained from venous blood at initial diagnosis were collected. All samples were analyzed within 2 h after collection using a Sysmex XN-9000 fully automated hematology analyzer.

The platelet-related parameters collected included: PLT (×10⁹/L), MPV (fL), PDW (%), plateletcrit (PCT, %), platelet-large cell ratio (P-LCR, %), IPF (%), and absolute reticulated platelet count (RP, ×10⁹/L).

### 2.5 Statistical analysis

Statistical analyses were performed using SPSS version 26.0 (IBM Corp., Armonk, NY, USA) and R software version 4.1. Continuous variables were assessed for normality using the Shapiro–Wilk test. Normally distributed data were expressed as mean ± standard deviation (mean ± SD) and compared between groups using the independent-samples *t* test. Non-normally distributed data were presented as median (interquartile range) [Median (IQR)] and compared using the Mann–Whitney *U* test. Categorical variables were expressed as number (percentage) [*n* (%)] and compared using the chi-square test or Fisher's exact test, as appropriate. Multicollinearity among independent variables was assessed by calculating the variance inflation factor (VIF), with a VIF < 5 indicating the absence of significant multicollinearity.

To identify independent predictors of progression to chronic immune thrombocytopenia, variables with *P* < 0.05 in univariate analysis were entered into a multivariate binary logistic regression model. Results were reported as adjusted odds ratios with corresponding 95% confidence intervals.

Receiver operating characteristic (ROC) curve analysis was performed to evaluate the discriminative performance of individual predictors and the combined predictive model, and the area under the curve (AUC) was calculated. Based on the final multivariate regression model, a visualized nomogram was constructed for individualized risk prediction. Internal validation of the predictive model was performed using bootstrap resampling with 1,000 iterations to evaluate model stability and estimate optimism-corrected performance. Both discrimination (AUC) and calibration were assessed after bootstrap correction. Calibration curves were generated to evaluate the agreement between predicted and observed probabilities. Decision curve analysis (DCA) was conducted to determine the clinical net benefit of the predictive model across a range of threshold probabilities. All statistical tests were two-sided, and a *P* value <0.05 was considered statistically significant.

## 3 Results

### 3.1 Comparison of baseline characteristics

A total of 167 children with newly diagnosed ITP were included in the present study. Based on follow-up outcomes, patients were classified into non-CITP (*n* = 136) and CITP groups (*n* = 31). No significant differences were observed between the two groups regarding sex distribution (*P* = 0.741) or BMI (*P* = 0.190), indicating good comparability of these baseline characteristics. Furthermore, no significant association was found between the initial treatment methods (IVIG, corticosteroids, TPO-RAs) and the progression to chronic immune thrombocytopenia. However, the mean age at diagnosis was significantly higher in the CITP group than in the non-CITP group, suggesting that age may be associated with the risk of progression to CITP (Table 1).

**Table 1:** Baseline characteristics of patients.

Variables	Non-CITP group (*n* = 136)	CITP group (*n* = 31)	*t*/*χ*^2^	*P*
Age (years)	5.92 ± 2.96	8.29 ± 2.98	−4.021	<0.001
Sex			0.109	0.741
Male (*n*/%)	79 (58.1%)	17 (54.8%)		
Female (*n*/%)	57 (41.9%)	14 (45.2%)		
BMI (kg/m^2^)	16.51 ± 1.46	16.90 ± 1.47	−1.316	0.190
IVIG (*n*/%)	51 (42.6%)	15 (48.4%)	0.362	0.547
Corticosteroid (*n*/%)	91 (66.9%)	23 (74.2%)	0.615	0.433
TPO-RAs (*n*/%)	5 (3.7%)	2 (6.5%)	0.454	0.500

BMI, body mass index; CITP, chronic immune thrombocytopenia.

### 3.2 Comparison of platelet-related parameters at initial diagnosis

Platelet-related parameters at initial diagnosis were compared (Table 2). Several platelet-related indices were distinctly differed. The PLT at diagnosis was lower in the CITP group (*P* < 0.001). In contrast, MPV and IPF were both higher in the CITP group (all *P* < 0.05). No statistically significant differences were detected between the two groups in terms of PDW, PCT, RP, or P-LCR (all *P* > 0.05). These findings suggest that a lower PLT and higher MPV and IPF at initial diagnosis may represent potential factors associated with the progression of pediatric ITP to chronic disease.

**Table 2:** Comparison of platelet-related parameters at initial diagnosis.

Variables	Non-CITP group (*n* = 136)	CITP group (*n* = 31)	*t*/*χ*^2^	*P*
PLT (×10⁹/L)	30.61 ± 10.32	20.14 ± 7.28	5.348	<0.001
MPV (fL)	11.55 ± 0.83	11.87 ± 0.80	−1.996	0.048
PDW%	18.95 ± 2.79	19.63 ± 2.08	−1.534	0.130
PCT%	0.10 ± 0.05	0.12 ± 0.16	−0.805	0.427
RP (×10⁹/L)	14.42 ± 4.96	14.47 ± 4.16	−0.054	0.957
IPF%	14.21 ± 6.43	17.47 ± 6.14	−2.571	0.011
P-LCR%	41.51 ± 12.46	43.43 ± 13.09	−0.763	0.446

PLT, platelet count; MPV, mean platelet volume; PDW, platelet distribution width; PCT, plateletcrit; RP, reticulated platelet count; IPF, immature platelet fraction; P-LCR, platelet-large cell ratio.

### 3.3 Multivariate logistic regression analysis for the risk of CITP

To identify independent predictors of progression to CITP, variables showing statistical significance in univariate analyses (age, PLT, MPV, and IPF) were entered into a multivariate logistic regression model. Multicollinearity diagnostics confirmed that there was no significant collinearity among the variables. The VIF values ranged from 1.006 to 1.037, indicating the absence of significant multicollinearity. The results of the multivariate logistic regression analysis are summarized in Table 3. As shown in Table 3, age at diagnosis and IPF% were independently associated with an increased risk of progression to CITP, whereas higher PLT at diagnosis was associated with a decreased risk. MPV was not a significant predictor in the multivariate model. These findings indicate that older age, lower platelet count, and higher immature platelet fraction at diagnosis are important risk factors for the development of chronic ITP.

**Table 3:** Multivariate logistic regression analysis for CITP.

Variable	*B*	SE	*W*	*P*	OR	95% CI	VIF
Age	0.285	0.091	9.889	0.002	1.330	1.113–1.588	1.036
PLT	−0.149	0.034	18.913	<0.001	0.862	0.806–0.922	1.006
MPV	0.436	0.317	1.892	0.169	1.546	0.831–2.876	1.037
IPF%	0.093	0.041	5.098	0.024	1.097	1.012–1.19	1.014

PLT, platelet count; MPV, mean platelet volume; IPF, immature platelet fraction.

### 3.4 Predictive performance of platelet-related parameters for CITP

**Figure 1:**
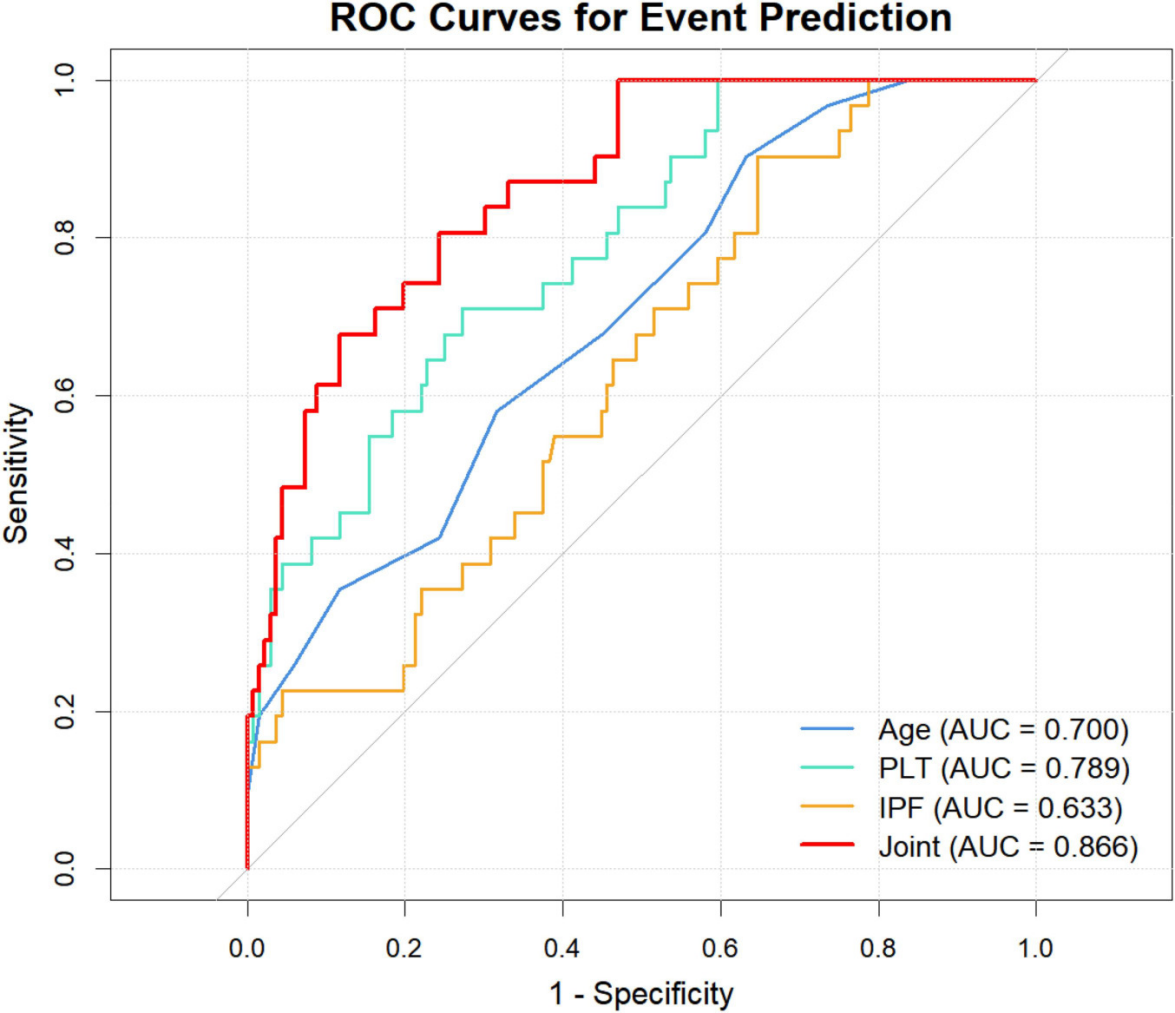
ROC curves for predicting the risk of CITP in children with ITP.

To further evaluate the discriminative ability of each independent predictor, ROC curve analyses were performed. As shown in Table 4 and Figure 1, among the individual predictors, PLT at initial diagnosis demonstrated the highest predictive performance (AUC = 0.789; 95% CI 0.706–0.872; *P* < 0.001). The optimal cutoff value for PLT was 22.734 × 10⁹/L. Age at diagnosis (AUC = 0.700; 95% CI 0.602–0.798) and IPF (AUC = 0.633; 95% CI 0.531–0.734) also showed statistically significant discriminative ability. A combined predictive model integrating age, PLT, and IPF exhibited significantly superior performance compared with any single parameter (AUC = 0.866; 95% CI: 0.797–0.936, *P* < 0.001). These results indicate that the combined assessment of age, PLT, and IPF enables more effective identification of children at risk of chronic ITP at the time of initial diagnosis, and that the optimal cutoff values of these parameters provide quantitative guidance for clinical risk stratification.

**Table 4:** ROC analysis of initial diagnostic indicators for predicting CITP in children.

Variables	AUC	SE	95% CI	*P*	Optimal cut-off value
Age	0.700	0.050	0.602–0.798	<0.001	4.5
PLT	0.789	0.042	0.706–0.872	<0.001	22.734
IPF%	0.633	0.052	0.531–0.734	0.001	10.415
Joint	0.866	0.035	0.797–0.936	<0.001	

PLT, platelet count; IPF, immature platelet fraction.

### 3.5 Construction of a nomogram for predicting the risk of CITP in children with newly diagnosed ITP

**Figure 2:**
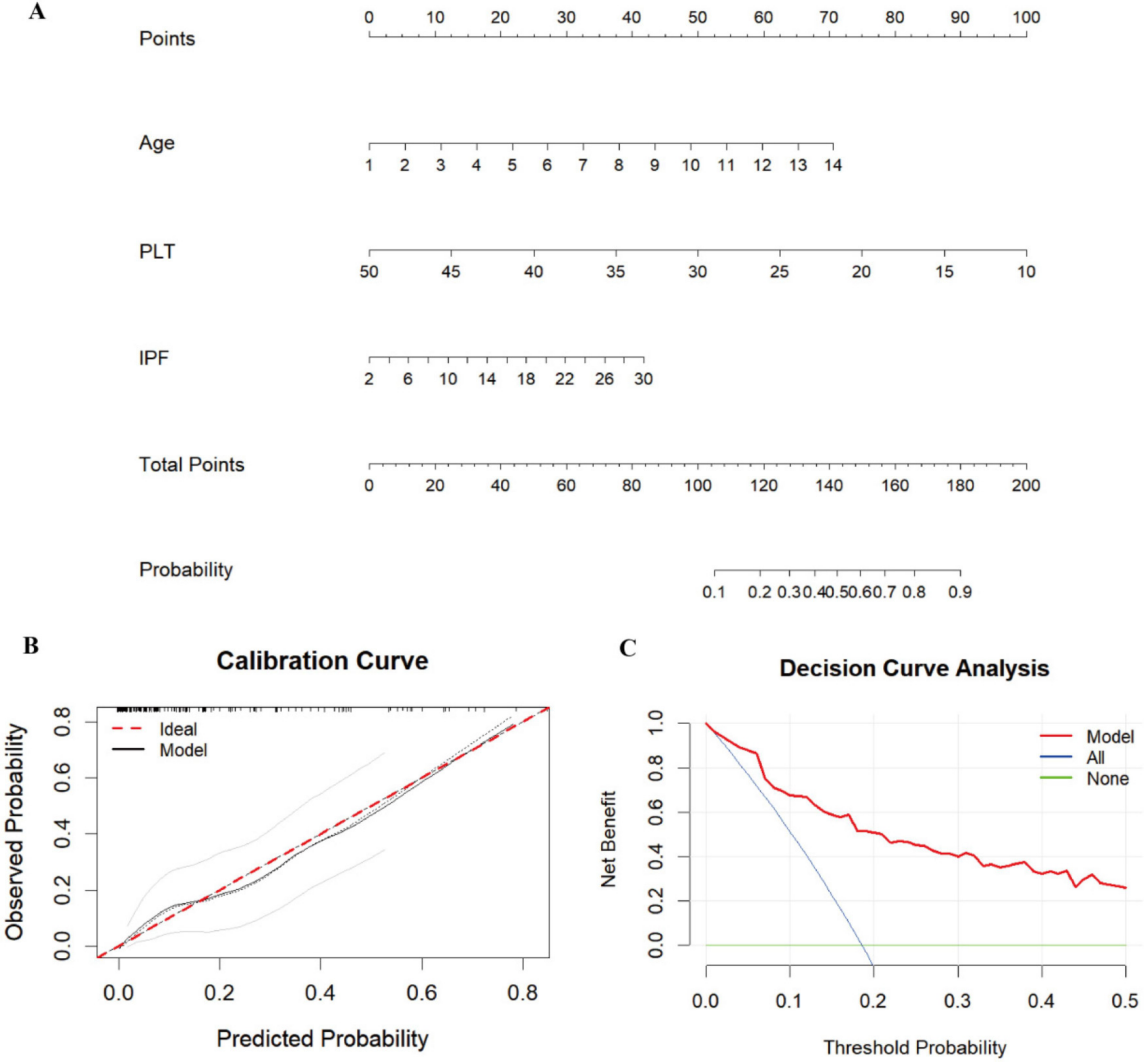
Predictive model for the risk of CITP in children with ITP. **(A)** Nomogram for predicting the risk of CITP in children with ITP. **(B)** Calibration curve of the predictive model for CITP risk. **(C)** DCA of the predictive model for CITP risk.

Age at diagnosis, PLT, and IPF were applied to develop a nomogram for individualized prediction of CITP risk in children with newly diagnosed ITP (Figure 2A). The model demonstrated good calibration, with close agreement between predicted and observed probabilities (Figure 2B), a mean absolute error of 0.019, and no significant difference in the Hosmer–Lemeshow test (*χ*^2^ = 9.429, *P* = 0.3074). Internal validation using bootstrap resampling (1,000 iterations) showed good model stability, with an optimism-corrected AUC of 0.856 compared with an apparent AUC of 0.866, indicating minimal overfitting. The calibration slope after correction was 0.942. Decision curve analysis (Figure 2C) demonstrated that the nomogram provided greater net benefit than treat-all or treat-none strategies within a threshold probability range of 0.05–0.50. These findings indicate that the nomogram exhibits favorable predictive accuracy, calibration, and clinical applicability, supporting its use for early identification of high-risk children and the implementation of individualized management strategies at initial diagnosis.

## 4 Discussion

This retrospective study demonstrated that among children with newly diagnosed ITP, older age at diagnosis, lower PLT, and higher IPF at initial presentation were independent risk factors for progression to CITP. The combined predictive model and nomogram constructed based on these three indicators exhibited favorable discriminative performance and clinical utility, providing a quantitative tool for early identification of children at high risk of chronic disease.

Consistent with previous classic studies and recent research results, our findings also indicate that older age at diagnosis is an independent predictor of chronic progression of childhood ITP. In a large prospective study, Kühne et al. clearly reported that age older than 10 years was one of the most important clinical predictors of chronic ITP (15). Reid further suggested that with increasing age, the immune system may respond to triggering events in a manner more prone to sustaining autoimmune activity, thereby increasing the likelihood of chronic disease (16). Our study further refines the clinical relevance of age by quantifying its predictive contribution within an integrated risk model, offering a more practical reference for early clinical assessment.

With regard to laboratory parameters, previous studies have suggested that more severe thrombocytopenia at initial diagnosis may reflect a more intense or less self-limiting autoimmune platelet destruction process (17). In the present study, PLT at diagnosis demonstrated the highest predictive performance among individual indicators, with a cutoff value as low as 22.7 × 10⁹/L indicating a markedly increased risk of chronicity. However, the predictive value of other platelet volume–related parameters remains controversial. Some studies have reported that increased MPV is associated with chronic progression (18, 19), whereas others have suggested that IPF may be more informative (20). In our study, MPV showed a significant association with CITP in univariate analysis. However, it did not retain independent predictive value in the multivariate model. This result likely has a specific meaning. MPV measures average platelet size. This measurement is influenced by platelet age, activation state, and production dynamics. The information it provides may be captured or superseded by IPF when both are considered together in a multivariate analysis. IPF reflects real-time thrombopoietic activity more directly and specifically. It does this by quantifying the proportion of newly released, immature platelets. Therefore, when assessed together with IPF and other key variables, MPV may not add extra independent prognostic information about disease chronicity.

One of the most important findings of this study is the identification of elevated IPF at initial diagnosis as an independent predictor of CITP. IPF directly quantifies the proportion of newly released, immature platelets in circulation and serves as a real-time indicator of compensatory megakaryopoiesis in the bone marrow. Previous studies have shown that elevated IPF may be observed in adults and children with persistent or chronic ITP, reflecting sustained compensatory platelet production (21). In the present study, IPF was further extended to the early diagnostic stage and showed a significant association with disease chronicity. However, when evaluated as a single marker, its discriminative performance was only moderate (AUC = 0.633), suggesting that it is not a strong standalone predictor. This is likely because IPF primarily reflects platelet production dynamics rather than the balance between immune-mediated destruction and clinical disease progression.Importantly, despite its limited individual predictive ability, IPF provided complementary biological information on platelet turnover that was not captured by platelet count or age. When incorporated into the multivariable model, it contributed to improved overall risk stratification performance, supporting its role as a supplementary rather than primary predictive factor. This pattern differs from the transient compensatory response followed by rapid recovery typically observed in acute, self-limiting ITP. By integrating age, PLT, and IPF, representing clinical characteristics, degree of platelet destruction, and bone marrow compensatory capacity, respectively, the combined model achieved superior performance.

By integrating age, PLT, and IPF, representing clinical characteristics, degree of platelet destruction, and bone marrow compensatory capacity, respectively, the combined model achieved superior performance. This highlights the advantage of multidimensional assessment in predicting complex disease outcomes. To facilitate clinical application, we further developed a nomogram, which demonstrated good calibration and clinically meaningful net benefit on DCA, in line with the current trend toward individualized and precision medicine. They provide clinicians with a simple, evidence-based tool to stratify patients at diagnosis according to their risk of developing chronic disease. This early risk assessment can inform several aspects of management: it may guide the intensity and frequency of follow-up, with higher-risk patients warranting closer monitoring; it can aid in patient and family counseling by setting realistic expectations regarding disease course; and it could potentially help in tailoring therapeutic strategies, though this requires further prospective investigation. The use of readily available clinical (age) and routine laboratory (PLT, IPF) parameters enhances the model's practicality for implementation in diverse clinical settings, aligning with the move toward individualized and precision medicine in pediatric hematology.

Several limitations of this study should be acknowledged. First, as a single-center retrospective study, it is subject to inherent selection bias. Second, although patients were managed according to current pediatric ITP guidelines, treatment strategies were individualized based on clinical condition, which may have introduced residual confounding. Third, the relatively small sample size of the CITP group may have limited the statistical power of certain subgroup analyses and affected the stability of model parameters. External validation in multicenter, large-scale prospective cohorts is required to confirm the generalizability of the model. In addition, this study did not incorporate more in-depth immunological parameters, such as specific viral loads, autoantibody profiles, or cytokine signatures. Integrating these factors with the current indicators may further enhance predictive performance and represents an important direction for future research.

## 5 Conclusion

This study demonstrates that combined assessment of age at diagnosis, PLT, and IPF effectively predicts the risk of chronic progression in children with newly diagnosed ITP. The nomogram developed and preliminarily validated in this study exhibits good discrimination, calibration, and clinical applicability, providing an evidence-based and practical tool for early risk stratification, optimization of follow-up strategies, and exploration of individualized intervention timing.

## Data Availability

The original contributions presented in the study are included in the article/Supplementary Material, further inquiries can be directed to the corresponding author.

## References

[1] Gotesman M Shear M Raheel S Procassini M Panosyan EH. Pediatric immune thrombocytopenia. Adv Pediatr. (2024) 71(1):229–40. 10.1016/j.yapd.2024.02.007 38944486

[2] Gellasch P Torraca M Okun ML. Sleep and mood among women with histories of depression when they used a responsive infant bassinet during the COVID-19 pandemic. J Obstet Gynecol Neonatal Nurs. (2024) 53(4):406–15. 10.1016/j.jogn.2024.02.006 38552674

[3] Mathias SD Li X Eisen M Carpenter N Crosby RD Blanchette VS. A phase 3, randomized, double-blind, placebo-controlled study to determine the effect of romiplostim on health-related quality of life in children with primary immune thrombocytopenia and associated burden in their parents. Pediatr Blood Cancer. (2016) 63(7):1232–7. 10.1002/pbc.25984 27037553 PMC5071741

[4] Mareddy C Kalra M Sachdeva A. Generic romiplostim for children with persistent or chronic immune thrombocytopenia: experience from a tertiary care centre in north India. Br J Haematol. (2022) 197(5):618–26. 10.1111/bjh.18126 35467751

[5] Kim CY Lee EH Yoon HS. High remission rate of chronic immune thrombocytopenia in children: result of 20-year follow-up. Yonsei Med J. (2016) 57(1):127–31. 10.3349/ymj.2016.57.1.127 26632392 PMC4696943

[6] Wang YZ Liu ZR. Relationship between the age of diagnosis and clinical outcomes in children with chronic immune thrombocytopenia. Zhongguo Shi Yan Xue Ye Xue Za Zhi. (2024) 32(4):1201–6. 10.19746/j.cnki.issn.1009-2137.2024.04.035 39192420

[7] Yan M Zhang Y Yang F Ji L Wang M Wang W. Comparative study between chronic immune thrombocytopenia patients and healthy population on Epstein–Barr virus infection status by polymerase chain reaction. Expert Rev Hematol. (2020) 13(7):781–6. 10.1080/17474086.2020.1772746 32498632

[8] Stasi R Willis F Shannon MS Gordon-Smith EC. Infectious causes of chronic immune thrombocytopenia. Hematol Oncol Clin North Am. (2009) 23(6):1275–97. 10.1016/j.hoc.2009.08.009 19932434

[9] Pinna A Porcu T Paliogiannis P Dore S Serra R Boscia F. Complete blood cell count measures in retinal artey occlusions. Acta Ophthalmol. (2021) 99(6):637–43. 10.1111/aos.14699 33629472

[10] Huang Z Liu WJ Guo QL Liu CY. Platelet parameters and expression of platelet membrane glycoprotein in childhood acute lymphoblastic leukemia. Genet Mol Res. (2015) 14(4):16074–89. 10.4238/2015.December.7.20 26662400

[11] Liu WJ Bai J Guo QL Huang Z Yang H Bai YQ. Role of platelet function and platelet membrane glycoproteins in children with primary immune thrombocytopenia. Mol Med Rep. (2016) 14(3):2052–60. 10.3892/mmr.2016.5504 27431926 PMC4991683

[12] Budak YU Polat M Huysal K. The use of platelet indices, plateletcrit, mean platelet volume and platelet distribution width in emergency non-traumatic abdominal surgery: a systematic review. Biochem Med. (2016) 26(2):178–93. 10.11613/BM.2016.020 PMC4910273 27346963

[13] World Medical Association. World medical association declaration of Helsinki: ethical principles for medical research involving human subjects. J Am Med Assoc. (2013) 310(20):2191–4. 10.1001/jama.2013.281053 24141714

[14] Neunert C Terrell DR Arnold DM Buchanan G Cines DB Cooper N. American Society of Hematology 2019 guidelines for immune thrombocytopenia. Blood Adv. (2019) 3(23):3829–66. 10.1182/bloodadvances.2019000966 31794604 PMC6963252

[15] Kuhne T Buchanan GR Zimmerman S Michaels LA Kohan R Berchtold W. A prospective comparative study of 2540 infants and children with newly diagnosed idiopathic thrombocytopenic purpura (ITP) from the intercontinental childhood ITP study group. J Pediatr. (2003) 143(5):605–8. 10.1067/s0022-3476(03)00535-3 14615730

[16] Reid MM. Chronic idiopathic thrombocytopenic purpura: incidence, treatment, and outcome. Arch Dis Child. (1995) 72(2):125–8. 10.1136/adc.72.2.125 7702373 PMC1511010

[17] Kasser S Bizeul A Chitlur M Donato H Roganovic J Vogt JE. Predicting chronicity in children and adolescents with newly diagnosed immune thrombocytopenia at the timepoint of diagnosis using machine learning-based approaches. Pediatr Blood Cancer. (2026) 73:e70103. 10.1002/1545-5017.70103 41524547

[18] Bowles KM Cooke LJ Richards EM Baglin TP. Platelet size has diagnostic predictive value in patients with thrombocytopenia. Clin Lab Haematol. (2005) 27(6):370–3. 10.1111/j.1365-2257.2005.00726.x 16307537

[19] Arshad A Mukry SN Shamsi TS. Clinical relevance of extended platelet indices in the diagnosis of immune thrombocytopenia. Acta Clin Croat. (2021) 60(4):665–74. 10.20471/acc.2021.60.04.14 35734488 PMC9196221

[20] Adly AA Ragab IA Ismail EA Farahat MM. Evaluation of the immature platelet fraction in the diagnosis and prognosis of childhood immune thrombocytopenia. Platelets. (2015) 26(7):645–50. 10.3109/09537104.2014.969220 25350586

[21] Jeon K Kim M Lee J Lee JS Kim HS Kang HJ. Immature platelet fraction: a useful marker for identifying the cause of thrombocytopenia and predicting platelet recovery. Medicine. (2020) 99(7):e19096. 10.1097/MD.0000000000019096 32049816 PMC7035018

